# Novel Method for Isolation of Murine Clara Cell Secretory Protein-Expressing Cells with Traces of Stemness

**DOI:** 10.1371/journal.pone.0043008

**Published:** 2012-08-16

**Authors:** Xiao-Yang Wang, Kathleen M. Keefe, Sandra M. Jensen-Taubman, Danlei Yang, Kai Yan, R. Ilona Linnoila

**Affiliations:** Cell and Cancer Biology Branch, Center for Cancer Research, National Cancer Institute, National Institutes of Health, Bethesda, Maryland, United States of America; Cincinnati Children’s Hospital Medical Center, United States of America

## Abstract

Clara cells are non-ciliated, secretory bronchiolar epithelial cells that serve to detoxify harmful inhaled substances. Clara cells also function as stem/progenitor cells for repair in the bronchioles. Clara cell secretory protein (CCSP) is specifically expressed in pulmonary Clara cells and is widely used as a Clara cell marker. In addition CCSP promoter is commonly used to direct gene expression into the lung in transgenic models. The discovery of CCSP immunoreactivity in plasma membranes of airway lining cells prompted us to explore the possibility of enriching Clara cells by flow cytometry. We established a novel and simple method for the isolation of CCSP-expressing cell Clara cells using a combination of mechanical and enzymatic dissociation followed by flow cytometry sorting technology. We showed that ∼25% of dissociated cells from whole lung expressed CCSP. In the resulting preparation, up to 98% of cells expressed CCSP. Notably, we found that several common stem cell markers including CD44, CD133, Sca-1 and Sox2 were expressed in CCSP^+^ cells. Moreover, CCSP^+^ cells were able to form spheroid colonies *in vitro* with 0.97‰ efficiency. Parallel studies *in vivo* confirmed that a small population of CCSP^−^expressing cells in mouse airways also demonstrates stem cell-like properties such as label retention and harboring rare bronchioalveolar stem cells (BASCs) in terminal bronchioles (TBs). We conclude that CCSP^+^ cells exhibit a number of stem cell-like features including stem cell marker expression, bronchosphere colony formation and self-renewal ability. Clara cell isolation by flow cytometry sorting is a useful method for investigating the function of primary Clara cells in stem cell research and mouse models.

## Introduction

Human lungs are composed of three functional and morphological compartments: proximal and distal airways and the alveolar compartment. Proximal airways are lined by a pseudostratified epithelium with a number of cell types with important protective functions such as ciliated cells, goblet cells, and basal cells. More distally, the lining is a simplified columnar epithelium largely made up of non-ciliated secretory cells called Clara cells, and a few ciliated and basal cells. [1], [2]. Further down, the respiratory bronchioles are lined by cuboidal epithelium comprised entirely of ciliated and Clara cells, whereas, the epithelium of the alveolar compartment is comprised of type I and type II cells. In mouse, the pseudostratified epithelium is limited to trachea and extrapulmonary main bronchi while Clara cells make up over 80% of the epithelium, with few interspersed ciliated cells, that line intrapulmonary conducting airways [3]. These features make mouse an excellent tool for studying the functions of Clara cells.

Clara cells have several protective properties. They detoxify xenobiotics and oxidant gasses, control inflammation, participate in mucociliary clearance of environmental agents, and proliferate/differentiate to maintain the ciliated and non-ciliated cell population. Clara cells are a source of cytochrome P450 enzymes that contribute to the metabolism of a variety of substances [4]. In addition to the major Clara cell secretory protein (CCSP), also known as CC10, CC16, Clara cell antigen, secretoglobin 1A1 (SCGB1A1) or uteroglobin, Clara cells also contribute surfactant apoproteins A, B and D, proteases, anti-microbial peptides, several cytokines and chemokines, and mucins in the extracellular fluid lining airspaces. CCSP is the most abundant secretory protein found in the airway surface fluid, expressed exclusively in non-ciliated Clara cells and widely used as a marker of the cells [5], [6], [7], [8].Changes in CCSP levels have a profound impact on not only the composition of airway surface fluid but also the airway epithelial response to environmental stimuli [9], [10]. Another important property of Clara cells is their ability to serve as progenitors for airway lining cells in response to injury. Moreover, subpopulations of CCSP-expressing cells may function as true stem cells of adult airways. Presently it is not known whether the groups overlap or represent distinct cells such as variant Clara cells [11], type A cells [12], OCT4-expressing stem cells [13] and bronchioalveolar stem cells (BASCs) [14].

Due to the lack of simple methods for the isolation of primary Clara cells from the lung, the majority of studies have been carried out *in vivo* or using lung cancer cells for *in vitro* tests. The major disadvantage of such approaches is the difficulty in performing mechanistic studies in non-neoplastic primary cells. Recently, Wong et al. developed a method for isolating CCSP^+^ cells from bone marrow by flow cytometry sorting [15]. We speculated that this method may also be used to isolate CCSP^+^ (Clara) cells from the lung. In this study we established a simple method for the isolation of CCSP^+^ cells from mouse lung and applied several different means to identify stem cell-like characteristics of CCSP^+^ cell *in vitro* and *in vivo*. We propose that this new procedure method for CCSP^+^ cell isolation provides a useful instrument for Clara cell research, for instance in the field of stem cell biology.

## Materials and Methods

### Mice

FVB mice were purchased from the Frederick National Lab, Maryland. Mice were housed under specific pathogen-free conditions under a 12-h light/dark cycle with access to food and water *ad libitum*. All the procedures used in this study were approved by the NIH Animal Care and Use Committee.

### Preparation of Single Cell Suspensions

**Figure 1:**
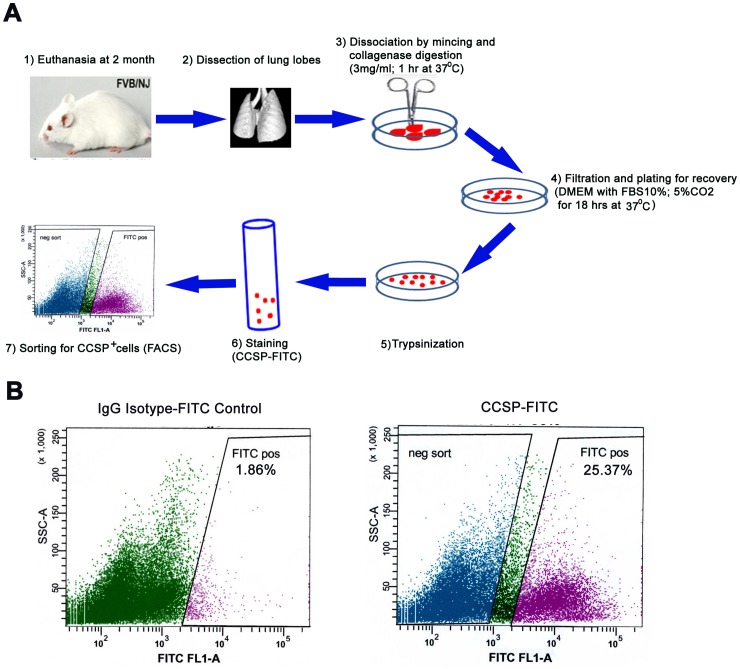
Schema for purification of primary CCSP positive cells from mouse lung. A) Two month old FVB mice were euthanized by CO_2,_ lungs removed and lobes collected. After washing in PBS, lobes were minced on ice and incubated in a small cell culture dish with 3 mg/ml collagenase in PBS (total 5 ml) in a shaking platform for 1 hour at 37°C. The suspension was further disaggregated by trituration through a 19 gauge needle, with 5 ml of PBS, filtered through a 40 µm cell strainer and centrifuged at 1000 rpm for 5 min. The supernatant was discarded, cells resuspended in red blood cell lysis buffer for 4 min re-plated into 10 cm culture dishes for recovery overnight (18 hrs). Surviving cells were adhering to the dish. After trypsinization and neutralization by 10% FBS media, cells were resuspended in PBS with 3% FBS, and stained with rabbit anti-CCSP antibody and FITC conjugated anti-rabbit secondary antibody. CCSP^+^ and CCSP^−^ cells were sorted with FACS Vantage SE cell sorter. B) Rabbit IgG was used as an isotype -matched negative control; CCSP^+^ population sorted with FACS Vantage SE cell sorter from dissociated lung tissue was 25.37%.

The heart, lungs and trachea were removed en bloc from mice following euthanasia by carbon dioxide inhalation. Lungs were separated and lobes minced on ice and incubated with collagenase type I (Invitrogen, Grand Island, NY) at 3 mg/ml in PBS in a volume of 2 ml per lung for 1 hour at 37°C with continuous agitation in an incubator. The suspension was further disaggregated by trituration through a 19 gauge needle (Sherwood Medical Co, St. Louis, MO), diluted in PBS. The crude cell suspension was filtered through a 40 µm cell strainer (BD Biosciences, Sparks, MD) and centrifuged at 700 rpm for 5 min. After discarding supernatant, cells were resuspended in 2 ml of red blood cell lysis buffer (eBioscience, San Diego, CA) for 4 min. Neutralization was performed with 10 ml of Dulbecco’s Modified Eagle Medium (DMEM) (Invitrogen) with 10% FBS (Invitrogen) and cells were centrifuged at 700 rpm for 5 min. Cells were resuspended in DMEM/10% FBS with 20 ng/ml gentamycin/0.5 ng/ml amphotericin B (Cascade Biologicas^tm^, Portland, Oregon), plated in 100 mm dishes and placed to recover in an incubator at 37°C and 5% CO_2_ for 18 hours (Figure 1A).

**Figure 2:**
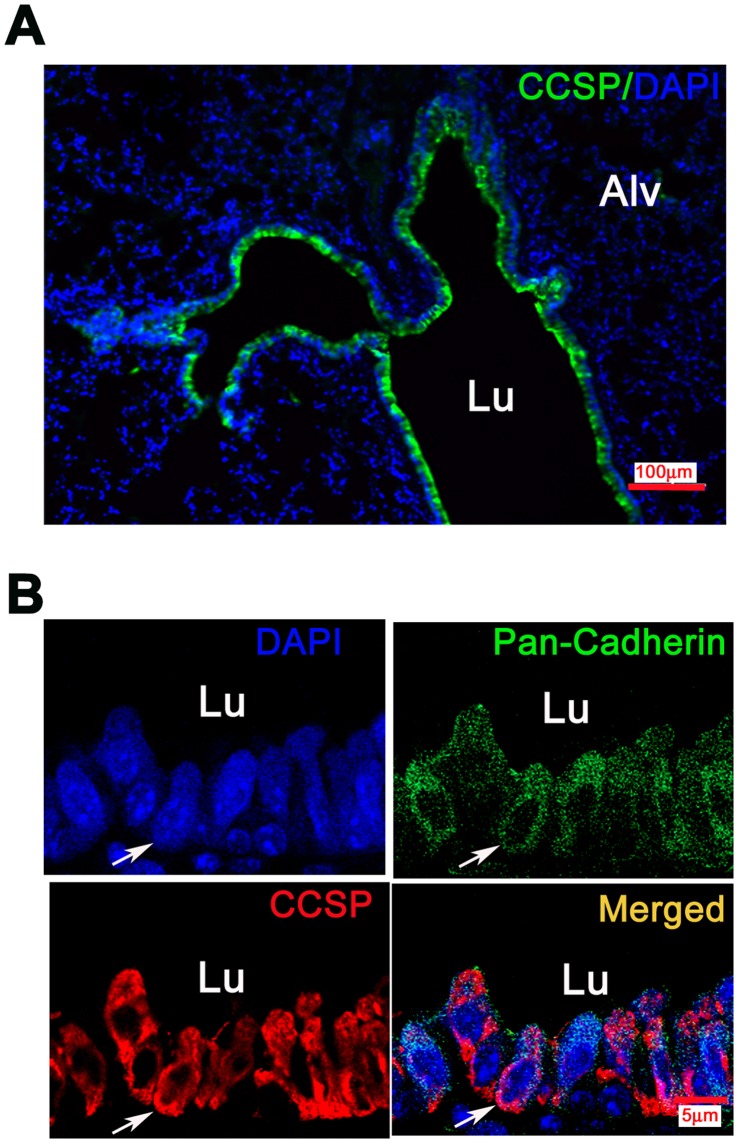
Membranous immunoreactiviy of CCSP in mouse airways. Photomicrographs of immunofluorescence in mouse lung tissue. A) Low power view of cells lining terminal bronchioles (TBs) that were intensely positive for CCSP (green fluorescence, Bar = 100 µm,Alv = alveoli) B) High power views of co-expression (yellow) of pan-cadherin (green) and CCSP (red) in airway epithelium. Pan-cadherin expression is along the cell membranes, CCSP in the cytoplasm and cell membranes, mostly on outer surface. Bar = 5 µm, Lu = lumen.

**Figure 3:**
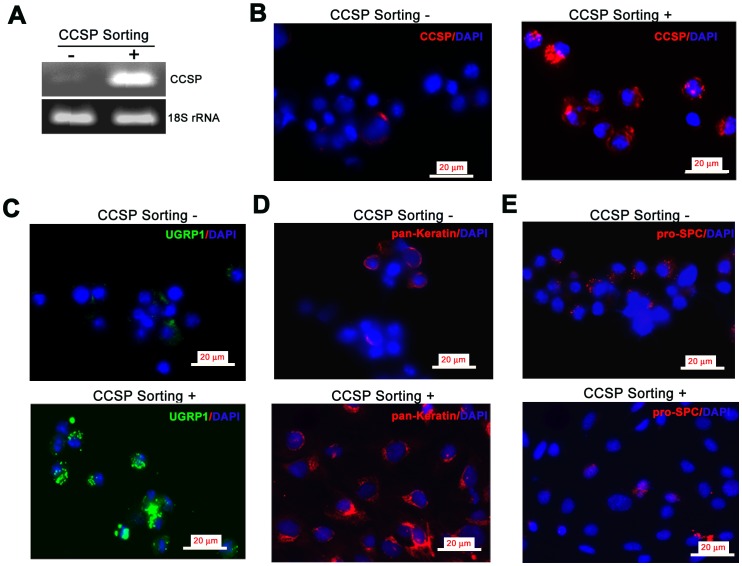
Characterization of sorted cells. A) RT-PCR of sorted cell fractions with minimal (CCSP^−^ fraction) and strong (CCSP^+^ fraction) expression of CCSP mRNA. B–E) Immunofluorescence staining of sorted cell fractions. B) CCSP (green fluorescence) was expressed in almost all cells of the CCSP^+^ fraction, but rarely in the CCSP^−^ fraction. C) Many cells were also positive for the Clara cell marker UGRP1 (green fluorescence) in CCSP^+^ fraction cells. D) Pan-keratin expression (red fluorescence) in CCSP^+^ cells confirmed that they were epithelial. Only small population of CCSP^−^ cells expressed pan-keratin. E) Few cells in either fraction expressed pro-SPC (red fluorescence). Nuclei appear blue (DAPI); Bar = 20 µm; CCSP Sorting negative = CCSP^−^ fraction; CCSP Sorting positive = CCSP^+^ fraction.

### Flow Cytometry

Recovered cells were trypsinized in 0.05% Trypsin-EDTA (Invitrogen) and resuspended at a concentration of 1×10^7^ cells in 100 µl PBS with 3% FBS. Two microliters of the rabbit anti-CCSP antibody (Millipore, Billerica, MA) was added, followed by a 30 min incubation on ice. Cells were washed twice in PBS with 3% FBS, then 2 µL goat anti-rabbit- FITC secondary antibody was added and incubated on ice for 30 min. After two washes in PBS with 3% FBS, cells were resuspended in the same but fresh media. Rabbit IgG staining was used as an isotype-matched negative control and CCSP staining with permeabilization of dissociated cells was used as a positive control. CCSP positive (CCSP^+^) and negative (CCSP^−^) fractions were obtained by fluorescence-activated cell sorting (FACS) using Vantage SE® cell sorter (BD, Bedford, MA). They were examined by immunfluorescence, adherent or 2D and sphere cell (3D) cultures and qRT-PCR.

**Figure 4:**
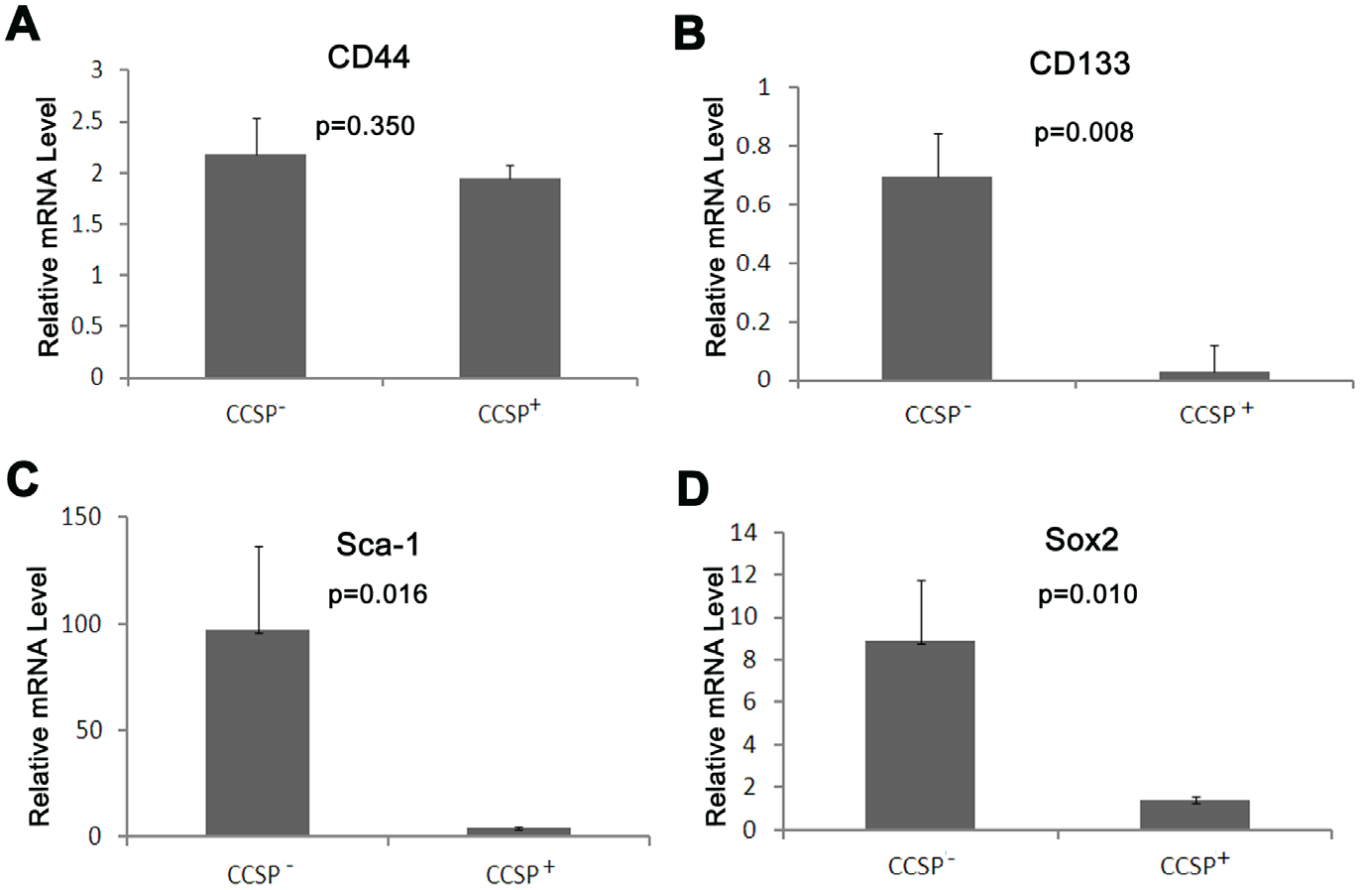
mRNA expression of stem cell markers in sorted cells. The mRNA of the stem cell markers CD44, CD133, Sca-1 and Sox2 was detected by qRT-PCR. A) The levels of CD44 mRNA were similar in CCSP^+^ and CCSP^−^ cells. B, C, D) Lower but still detectable CD133, Sca-1 and Sox2 mRNA levels in CCSP^+^ cells.

### Immunofluorescence and Immunohistochemistry

Single and dual labeling of cells and tissue sections by immunofluorescence (IF) or immunohistochemistry (IHC) was performed according to previously described methods [16]–[17]. The primary antibodies were: goat polyclonal anti-CCSP(T18) (1∶50, Santa Cruz Biotech, Santa Cruz, CA), rabbit anti- uteroglobin-related protein 1 (UGRP1) (1∶100, a kind gift from Dr. Shioko Kimura, NCI/NIH, Bethesda, MD), rabbit anti-pan-cadherin (, 1∶50, Abcam, Cambridge, MA), mouse anti-β-catenin (BD). rabbit anti-pan-cytokeratin (1∶100, Dako, Carpinteria, CA), rabbit anti-pro-SPC (1∶200, Millipore), rat anti-BrdU (1∶100, Accurate Chemical & Scientific Corp, Westbury, NY), rabbit anti-sox2(1∶2000, Seven Hill, Cincinnati, OH) and rabbit anti-ALDH1(1∶500. Abcam). Approximately 1×10^5^ FACS sorted cells, were washed twice in PBS with 1% FBS and resuspended in 30 µl of Cell Adherence Solution (Crystalgen, Commack, NY). After standing for 2 minutes, 3 µl of the cell mixture was mounted on glass slides, dried for 2 minutes and fixed with 4% paraformaldehyde in PBS for 15 minutes. Both fixed cells and tissue sections were blocked with 1% goat or rabbit normal serum for 1 hour. The blocking solution was removed and 75 µl of primary antibody was added to cells. After 1 hour of incubation at room temperature, slides were washed in PBS three times. Secondary antibodies were added. For dual-labeling IF, additional primary antibodies were added after the third PBS wash, followed by incubation with secondary antibodies conjugated with Alexa fluor 488 or 594 (Invitrogen). All incubations were performed at room temperature and slides were washed in PBS (3×5 min) between each step and mounted with an anti-fading reagent with 4′,6-diamidino-2-phenylindole (DAPI) (Invitrogen). Control slides were included in each analysis in which non-immune serum was substituted for primary antibodies and secondary antibodies individually. All IF images were taken with a Zeiss LSM 510 Meta Mk4 Confocal Microscope (Zeiss, Thornwood, New York). For IHC, signals were developed using 3,3′-diaminobenzidine (DAB).

**Figure 5:**
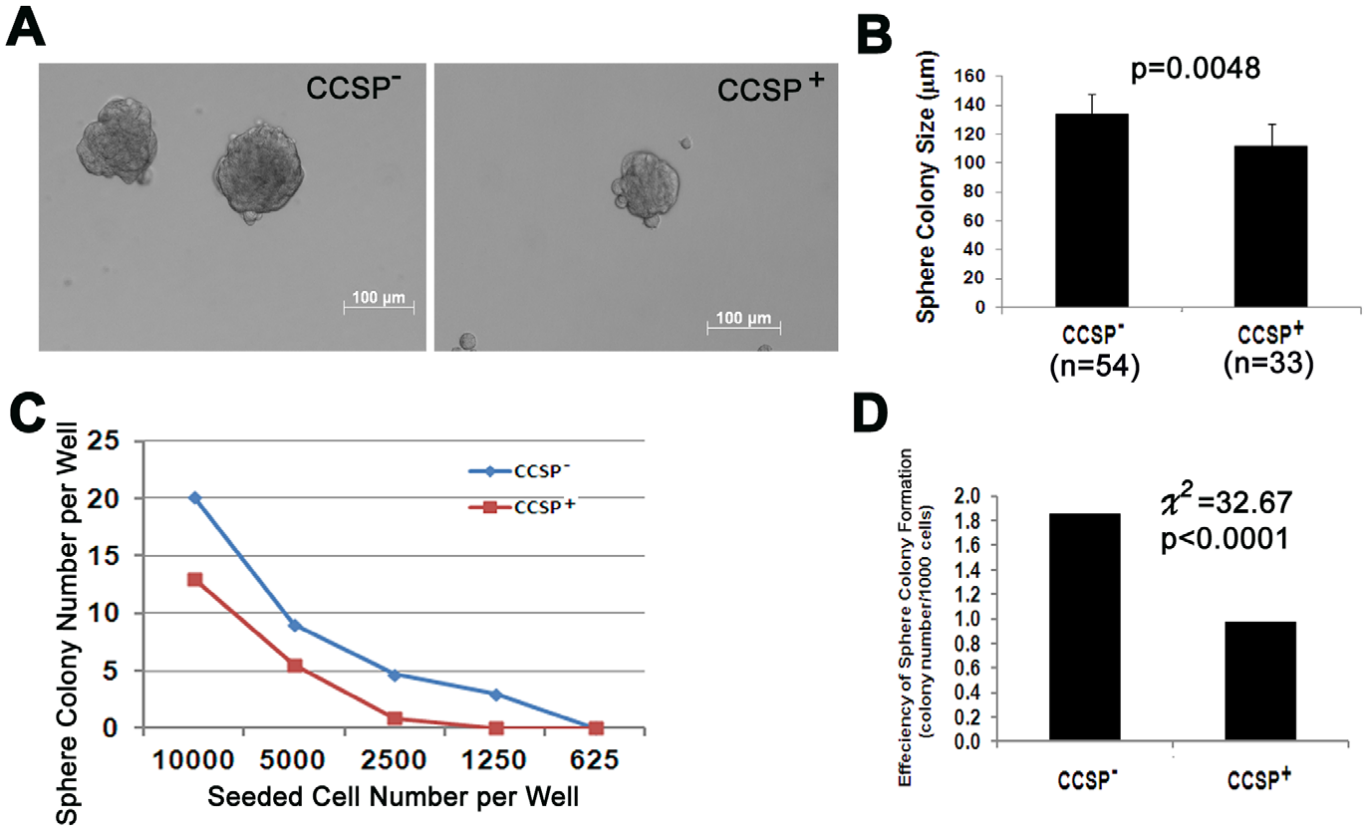
Bronchosphere formation by sorted cells. A) Phase-contrast photomicrographs of bronchospheres (Bar = 100 µm). B) The diameter of all spheroid colonies in the wells which 5000 cells were seeded. Bar graph reveals average spheroid colony size (µm). (Mean ±S.D. p = 0.0048, Student t-test). C) Average spheroid colony number per well in serial diluted cells after one week sphere cell culture. D) Efficiency of sphere colony formation = total colony number/total seeded cell ×1000. Efficiency of sphere colony formation was higher in CCSP^−^ cells than that in CCSP^+^ cells (p<0.0001, Chi-square test).

### Quantitative Real Time RT-PCR (qRT-PCR)

Total RNA from sorted cells was isolated using an RNeasy minikit (Qiagen, Valencia, CA) by following the manufacturer’s protocol. One microgram of RNA was reverse transcribed in a total volume of 20 µl using the QuantiTect RT kit (Qiagen). PCR was performed in triplicate in a MyiQ single color real time PCR detection system (Bio-Rad, Hercules, CA) using SYBR Green PCR kit (Qiagen) according to the manufacturer’s protocol. Amplification was confirmed by ethidium bromide staining of the PCR products on an agarose gel. The expression of each target gene was normalized to the expression of 18 S RNA and presented as the ratio of the target gene to 18 S RNA, expressed as 2^−ΔCt^, where Ct is the threshold cycle and ΔCt = Ct^Target^ − Ct^18S^. The primer sequences for qRT-PCR included: 5′-CACATATTGCTTCAATGCCTCAG-3′ (CD44 Forward), 5′-CCATCACGGTTGACAATAGTTATG-3 (CD44 Reverse), 5′-TGTTCTGGTTCGGCATAGGGAAAGCCAC-3′ (CD133 Forward), 5′-CTTGTCATAACAGGATTGTGAACACC -3′ (CD133 Reverse), 5′-GTCCCATTTGAGACTTCTTGCC-3′ (Sca-1Forward), 5′-AGGAGGGCAGATGGGTAAGC-3′ (Sca-1 Reverse), 5′-TGCTGCCTCTTTAAGACTAGGG-3′ (Sox2 Forward), 5′-TCGGGCTCCAAACTTCTC-3′ (Sox2 Reverse), 5′-TCGGAACTGAGGCCATGATT-3′ (18S forward), 5′-CCTCCGACTTTCGTTCTTGATTT-3′ (18S Reverse).

### Bronchosphere Cell Culture

FACS sorted cells were plated in 96-well ultralow attachment plates (Sigma-Aldrich, St. Louis, MO) at 10000, 5000, 2500,1250, 625, 313, 156, 78 viable cells/well in serum-free DMEM-F12 (Invitrogen) supplemented with 1×B27 supplement (Invitrogen), 20 ng/ml bFGF (Invitrogen), 20 ng/ml EGF (Invitrogen), 10 µg/ml insulin (Sigma-Aldrich) 10^−6^ M hydrocortisone (Sigma-Aldrich) and 20 ng/ml gentamycin/0.5 ng/ml amphotericin B. After 1 week, cell spheroid colony numbers were counted and colony size was measured under a Zeiss Axio Observer Z1 Inverted Microscope (Zeiss). Secondary sphere culture was performed after digestion of first sphere colonies by 0.05% Trypsin-EDTA (Invitrogen).

### Label Retention *in vivo*

For continuous labeling *in vivo*, BrdU (50 mg/ml) was administered to mice throughout a 7-day period via a subcutaneous miniosmotic pump (Alzet model 2001, Durect Corporation, Cupertino, CA). Alzet pumps were implanted in mice and removed after one week. Mice were sacrificed 4 weeks after removal of the Alzet pumps. Lungs were fixed overnight via tracheal instillation of fresh 4% paraformaldehyde and embedded in paraffin prior to sectioning. Label-retaining cells were identified by BrdU immunofluorescence. BrdU and CCSP double staining was performed and cells exhibiting a nucleus and attachment to basement membrane were counted. Bronchiolioles (BLs) were defined as intrapulmonary airways in which smooth muscle, but neither cartilage nor glands, could be seen. Terminal bronchioles (TBs) contained an intact bronchioalveolar duct junction (BADJ) and visible alveolar duct [17]. In TBs quantification of staining included all cells within 200 µm of the BADJ. A total 49 TBs and 26 BL structures were analyzed in lung sections of five mice.

**Figure 6:**
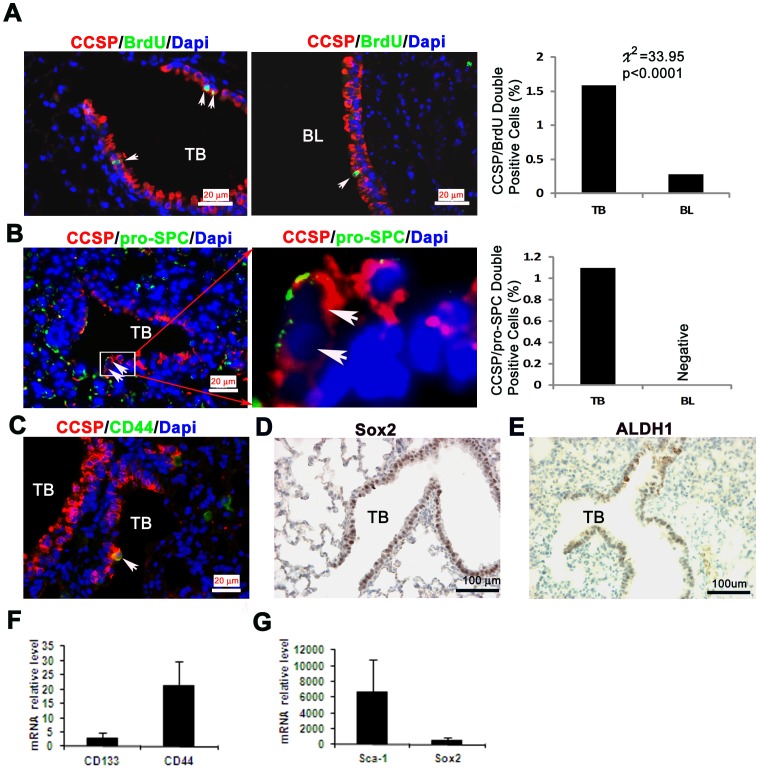
Evidence of stem cell features in mouse lung. A) Label retention by CCSP positive cells in airways. Double staining of BrdU and CCSP in the TB (left panel) and BL (middle panel). Barograph demonstrated the quantitative BrdU/CCSP double positive cells in TB and BL (IF, mouse number = 5, total TBs = 49, BLs = 26). B) Bronchioalveolar stem cells (BASCs) in the airway epithelium. CCSP and pro-SPC double immunofluorescence staining. Double positive cells were represented in TB (Left and middle panel, white arrow). Barograph showed the quantitative CCSP/Pro-SPC double positive cells in TB and BL. (IF, mouse number = 7, total TBs = 21, BLs = 20) C) A solitary double CD44/CCSP positive cell in a TB (immunofluorescence staining, bar = 20 µm). D) Sox2 nuclear (immunoperoxidase staining, bar = 100 µm), E) ALDH1 (immunperoxidase staining, bar = 100 µm) F-G) Bar graphs of qRT-PCR analyses for the relative expression of CD133, CD44, Sca-1 and Sox2 mRNAs in mouse lungs (mean±SD).

## Results

### CCSP Immunoreactivity is Discovered Along the Membranes of Clara Cells in Mouse Airways

It is well established that CCSP is expressed in non-ciliated Clara cells in the airways. CCSP which is widely used as a Clara cell marker is a cytoplasmic secretory protein [3]. Figure 2A revealed intense immunorectivity along the lining of mouse TB. Recently, Wong AP et al. was able to isolate CCSP^+^ cells from bone marrow using flow cytometry [15]. Therefore, we postulated that CCSP may be expressed not only in the cytoplasm, but also in the cell membrane of Clara cells. To obtain evidence for this, we used the well-known cell membrane marker pan-Cadherin [18]. Indeed, in high magnification photomicrographs we were able to demonstrate co-expression with pan-Cadherin in the cell membrane using confocal microscope (Figure 2B). These data suggested a possibility for isolating living Clara cells by flow cytometry sorting and lead us to develop the protocol outlined in this study.

### Isolation of CCSP^+^ Cells from Mouse Lung Using Fluorescence-activated Cell Sorting (FACS)

To test the possibility of CCSP^+^ cell isolation by flow cytometry from mouse lung, we established a simple method to make single cell suspensions from lung tissues. Using a combination of mincing by scissors and incubation in a high concentration of collagenase (3 mg/ml) for digestion, single cells were obtained within 2 hours from euthanasia. After an overnight recovery in DMEM/10%FBS cell culture media, cells adherent to culture dishes were trypsinized and sorted using a flow cytometry sorter. In FVB mice, about 25% of the lung cells sorted from one whole lung single cell suspension were CCSP^+^ (Figure 1). Typical yields of sorted cells per mouse were about ∼2.5×10^5^ of CCSP^+^ cells and ∼4×10^5^ of CCSP^−^ cells. Sorted cells were plated into 100 mm cell culture dish for overnight in an incubator prior to further studies. Unattached dead cells were removed with the media.

### Characterization of Sorted Cells

Sorted cells were used for RNA isolation and RT-PCR following an overnight recovery. CCSP mRNA expression was detected in CCSP^+^ cell fraction, but not in CCSP^−^ cells (Figure 3A). We also mounted cells on slides using Cell Adherence Media for immunofluorescence (IF). We found that 98% (225/230) of the cells in CCSP^+^ sorted fraction were positive for CCSP IF. We also found that 97% of the cells in the CCSP^+^ fraction revealed the expression of another Clara cell maker UGRP1 by IF. All of the cells in CCSP^+^ sorted fraction expressed pan-keratin. Rare CCSP^+^ sorted cells revealed the presence of pro-SPC (Figure 3). These data demonstrated that sorting by flow cytometry is a useful and simple method for harvesting purified CCSP-containing Clara cells.

### Expression of Stem Cell Markers

One of the features of stem/progenitor cells is the expression of stem cell markers. Therefore we performed qRT-PCR for several common stem cell markers including CD44, CD133, Sca-1 and SOX2 in the sorted cells. Interestingly, CD44 was expressed in both CCSP^+^ and CCSP^−^ populations at similar levels. In contrast, CD133, Sca-1 and Sox2 demonstrated much lower but detectable levels in CCSP^+^ cells than the levels in CCSP^−^ cells. These data indicate that CCSP^+^ cells express stem cell markers, although at low levels (Figure 4).

### Bronchosphere Formation

Spheroid culture is a common method to detect stem cell features *in vitro* [19]. Spheroid colony formation was tested by serial dilution technique. Both CCSP^+^ and CCSP^−^ cellular fractions were able to form sphere clones. However, following 10 days of culture (Figure 5) CCSP^-^ cells demonstrated a larger colony size and higher efficiency of colony formation than CCSP^+^ cells. Dissociation of spheroid colonies into single cells resulted in reformation of the spheroid colonies, indicating that this phenotype was stable (data not shown).

### BrdU Label Retention *in vivo* by CCSP^+^ Cells

Quiescent or slow-cycling stem cells in adult tissues can retain BrdU over long periods by either segregating chromosomes asymmetrically or dividing slowly. Label-retaining cells can be used to identify populations that contain stem cells [20]. In fact, many such studies have been used to determine putative stem cell locations in mammalian tissues [21], [22]. Using CCSP and BrdU double staining by IF, we found that 1.59% (39/2450) of cells in TBs and only 0.39% (12/4138) of them in BLs were BrdU^+^/CCSP^+^ (Figure 6). The results suggest that the majority of mouse airway CCSP^+^ stem/progenitor cells may reside in TBs.

### Evidence for Bronchioalveolar Stem Cells (BASCs) Mouse Airways

A subpopulation of CCSP^+^/pro-SPC^+^ cells known as bronchioalveolar stem cells (BASCs) are capable of differentiating into Clara cells and alveolar type II cells and are considered to be adult lung stem cells [14]. In the current study, a rare portion of sorted CCSP^+^ cells were also found to express the type II cell marker pro-SPC (Figure 3D). In order to confirm the existence of BASCs *in vivo*, we performed CCSP/pro-SPC double staining by IF in mouse lungs. Our results showed that 1.1% of TB epithelial cells contained BASCs while no CCSP^+^/pro-SPC^+^ double positive epitheliums were detected in BLs (Figure 6). In addition, a number of stem cell markers such as CD44, Sox2 and ALDH1 were detected by IF or IHC along the TB epithelium (Figure 6C–E). We also found that CD133, CD44, Sca-1 and Sox2 mRNAs were expressed at variable levels in mouse lung tissues (Figure 6F, 6G). This provides further evidence for the progenitor role that Clara cells may have in the mouse lung.

## Discussion

In this study, we isolated and characterized significantly purified CCSP-expressing cell populations from mouse lung by using high concentrations of collagenase and a flow cytometric sorting method. In addition, we showed that CCSP^+^ cells expressed stem cell markers and form three dimensional spheroid colonies in culture. Furthermore, we confirmed that CCSP^+^ cells may also express stemness characteristic *in vivo* as evidenced by label retention, the presence of CCSP/pro-SPC double positive BASCs and expression of stem cell markers in the epithelial lining of TBs of mice. Accordingly, the novel method described herein is a significant step in the progress of isolating and characterizing highly purified Clara cells in primary cultures.

Based on previous publications, the distribution of CCSP expression in non-ciliated Clara cells is described as cytoplasmic [23], [24], [25]. We made the surprising and novel discovery of CCSP immunoreactivity along cellular membranes of bronchiolar Clara cells. Using pan-cadherin as a cell membrane marker in normal airway epithelium [18], [26], [27] we found CCSP was expressed not only in the cytoplasm, but also in the membrane. These findings gave rise to the possibility that living Clara cells can be isolated by flow cytometry using fluorescing tags. Our successful CCSP^+^ cell sorting further confirmed the distribution of CCSP membranous expression. One explanation is that bronchiolar Clara cells secrete such large quantities of CCSP that part of it remains stuck to the outer surfaces of cell membranes, allowing sorting of CCSP-containing cells from suspension.

Clara cell isolation from rabbit was first reported in the early 1980s by Devereux et al. [28]. After that, several groups were able to isolate pulmonary Clara cells from mouse [29], [30], [31], [32], [33]. The studies have been instrumental in establishing the many functions of Clara cells. However, the majority of the methods are quite complex and rely on protease digestion followed by centrifugal elutriation and/or Percoll density gradient centrifugation. Only one group used FACS for Clara cell isolation from rat based on the reaction of their glutathione content with monochlorobimine to a fluorescent product [34]. The techniques typically resulted in a Clara cell enrichment of 55∼90%. A reproducible source of considerably purified Clara cells is necessary for airway stem/progenitor cell research. Using high concentrations of collagenase for lung tissue digestion followed by flow cytometry sorting, we were able to achieve 98% pure CCSP^+^ (Clara) cell population, providing a very useful and reliable method for Clara cell function and stem cell research. A notable application will be to directly address molecular mechanisms of genes that have been expressed in Clara cells by using CCSP as a lung specific promoter in transgenic mice.

To further characterize sorted CCSP^+^ cells, we evaluated the expression of pan-keratin protein in CCSP^+^ cells. All cells expressed pan-keratin indicating that all the CCSP^+^ cells were epithelial. We also found that a few cells expressed pro-SPC. This suggests that CCSP^+^ cells contain rare populations of BASCs (CCSP/SPC double positive cells).

In this study, the expression of well documented stem cell markers such as CD44 [35], CD133 [36], [37], Sca1 [14], [38] and Sox2 [39], [40] was detectable by qRT-PCR in CCSP^+^ cells. However, the level of CD133, Sca-1 and Sox2 expression was lower in CCSP^+^ cells than that in CCSP^−^ cells. One possible explanation is that CCSP is a Clara cell differentiation marker, so a CCSP^+^ population of cells may contain more mature Clara cells, but few stem/progenitor cells, while CCSP^−^ cells fraction is a mixture of many cells, such as type I, type II, ciliated cells, basal cells, smooth muscle cells and fibroblast cells and so on. Many of the cells have been shown to have stem cell features [19], [41]. Sphere culture showed that CCSP^+^ cells were able to form spheroid colonies. The sphere colony size and efficiency of colony formation were lower in CCSP^+^ cells compared to CCSP^−^ cells. This data further suggests that CCSP^+^ cells do have stem cell features, but stem cell activities are lower than in CCSP^−^ cells.

Using tissue sections, we found that 1.59% of CCSP positive cells in TBs were label-retaining cells and 1.1% were CCSP/SPC double positive BASCs. These data provide *in vivo* validation for the *in vitro* results that the CCSP^+^ cell population contains a small subset of stem cells in the airway.

In summary, we discovered that CCSP was not only expressed in the cytoplasm but there was also marked immunoreactivity along in the cell membranes of airway Clara cells. This provided the basis of flow cytometry sorting technology for the isolation of CCSP expressing Clara cells from murine lung. We also found that *in vitro*, CCSP^+^ cells demonstrated stem cell-like features including stem cell marker expression, bronchosphere colony formation and self-renewal ability. Moreover, a subset of label-retaining cells and BASCs were detectable in the CCSP^+^ population *in vivo* located in the TBs We conclude that Clara cell isolation by FACS is a useful method for investigating Clara cell function and overall pulmonary stem cell research biology.

## References

[1] Boers JE, Ambergen AW, Thunnissen FB (1999) Number and proliferation of clara cells in normal human airway epithelium. American Journal of Respiratory and Critical Care Medicine 159: 1585–1591. 10228131 10.1164/ajrccm.159.5.9806044

[2] Boers JE, Ambergen AW, Thunnissen FB (1998) Number and proliferation of basal and parabasal cells in normal human airway epithelium. American Journal of Respiratory and Critical Care Medicine 157: 2000–2006. 9620938 10.1164/ajrccm.157.6.9707011

[3] Wong AP, Keating A, Waddell TK (2009) Airway regeneration: the role of the Clara cell secretory protein and the cells that express it. Cytotherapy 11: 676–687. 19878054 10.3109/14653240903313974

[4] Stripp BR, Lund J, Mango GW, Doyen KC, Johnston C, et al (1996) Clara cell secretory protein: a determinant of PCB bioaccumulation in mammals. American Journal of Physiology 271: L656–664. 8897914 10.1152/ajplung.1996.271.4.L656

[5] Hackett BP, Shimizu N, Gitlin JD (1992) Clara cell secretory protein gene expression in bronchiolar epithelium. American Journal of Physiology 262: L399–404. 1566856 10.1152/ajplung.1992.262.4.L399

[6] Coppens JT, Van Winkle LS, Pinkerton K, Plopper CG (2007) Distribution of Clara cell secretory protein expression in the tracheobronchial airways of rhesus monkeys. Am J Physiol Lung Cell Mol Physiol 292: L1155–1162. 17237148 10.1152/ajplung.00454.2006

[7] Jensen-Taubman S, Wang XY, Linnoila RI (2010) Achaete-scute homologue-1 tapers neuroendocrine cell differentiation in lungs after exposure to naphthalene. Toxicological Sciences 117: 238–248. 20554700 10.1093/toxsci/kfq177 PMC2923285

[8] Linnoila RI, Szabo E, DeMayo F, Witschi H, Sabourin C, et al (2000) The role of CC10 in pulmonary carcinogenesis: from a marker to tumor suppression. Annals of the New York Academy of Sciences 923: 249–267. 11193761 10.1111/j.1749-6632.2000.tb05534.x

[9] Stripp BR, Reynolds SD, Boe IM, Lund J, Power JH, et al (2002) Clara cell secretory protein deficiency alters clara cell secretory apparatus and the protein composition of airway lining fluid. American Journal of Respiratory Cell and Molecular Biology 27: 170–178. 12151308 10.1165/ajrcmb.27.2.200200270c

[10] Stripp BR, Reynolds SD, Plopper CG, Boe IM, Lund J (2000) Pulmonary phenotype of CCSP/UG deficient mice: a consequence of CCSP deficiency or altered Clara cell function? Annals of the New York Academy of Sciences 923: 202–209. 11193758 10.1111/j.1749-6632.2000.tb05531.x

[11] Hong KU, Reynolds SD, Giangreco A, Hurley CM, Stripp BR (2001) Clara cell secretory protein-expressing cells of the airway neuroepithelial body microenvironment include a label-retaining subset and are critical for epithelial renewal after progenitor cell depletion. American Journal of Respiratory Cell and Molecular Biology 24: 671–681. 11415931 10.1165/ajrcmb.24.6.4498

[12] Evans MJ, Johnson LV, Stephens RJ, Freeman G (1976) Renewal of the terminal bronchiolar epithelium in the rat following exposure to NO2 or O3. Laboratory Investigation 35: 246–257. 957607

[13] Ling TY, Kuo MD, Li CL, Yu AL, Huang YH, et al (2006) Identification of pulmonary Oct-4+ stem/progenitor cells and demonstration of their susceptibility to SARS coronavirus (SARS-CoV) infection in vitro. Proceedings of the National Academy of Sciences of the United States of America 103: 9530–9535. 16772384 10.1073/pnas.0510232103 PMC1480441

[14] Kim CF, Jackson EL, Woolfenden AE, Lawrence S, Babar I, et al (2005) Identification of bronchioalveolar stem cells in normal lung and lung cancer. Cell 121: 823–835. 15960971 10.1016/j.cell.2005.03.032

[15] Wong AP, Keating A, Lu WY, Duchesneau P, Wang X, et al (2009) Identification of a bone marrow-derived epithelial-like population capable of repopulating injured mouse airway epithelium. Journal of Clinical Investigation 119: 336–348. 19164856 10.1172/JCI36882 PMC2631300

[16] Wang XY, Yin Y, Yuan H, Sakamaki T, Okano H, et al (2008) Musashi1 modulates mammary progenitor cell expansion through proliferin-mediated activation of the Wnt and Notch pathways. Molecular and Cellular Biology 28: 3589–3599. 18362162 10.1128/MCB.00040-08 PMC2423292

[17] Wang XY, Dakir el H, Naizhen X, Jensen-Taubman SM, DeMayo FJ, et al (2007) Achaete-scute homolog-1 linked to remodeling and preneoplasia of pulmonary epithelium. Laboratory Investigation 87: 527–539. 17507989 10.1038/labinvest.3700552

[18] Dai X, Galligan JJ (2006) Differential trafficking and desensitization of human ET(A) and ET(B) receptors expressed in HEK 293 cells. Exp Biol Med (Maywood) 231: 746–751. 16740992

[19] McQualter JL, Yuen K, Williams B, Bertoncello I (2010) Evidence of an epithelial stem/progenitor cell hierarchy in the adult mouse lung. Proceedings of the National Academy of Sciences of the United States of America 107: 1414–1419. 20080639 10.1073/pnas.0909207107 PMC2824384

[20] Fernandez-Gonzalez R, Illa-Bochaca I, Shelton DN, Welm BE, Barcellos-Hoff MH, et al (2010) In situ analysis of cell populations: long-term label-retaining cells. Methods in Molecular Biology 621: 1–28. 20405356 10.1007/978-1-60761-063-2_1

[21] Potten CS, Owen G, Booth D (2002) Intestinal stem cells protect their genome by selective segregation of template DNA strands. Journal of Cell Science 115: 2381–2388. 12006622 10.1242/jcs.115.11.2381

[22] Zhang J, Niu C, Ye L, Huang H, He X, et al (2003) Identification of the haematopoietic stem cell niche and control of the niche size. Nature 425: 836–841. 14574412 10.1038/nature02041

[23] Dave V, Wert SE, Tanner T, Thitoff AR, Loudy DE, et al (2008) Conditional deletion of Pten causes bronchiolar hyperplasia. American Journal of Respiratory Cell and Molecular Biology 38: 337–345. 17921358 10.1165/rcmb.2007-0182OC PMC2258453

[24] Katavolos P, Ackerley CA, Viel L, Clark ME, Wen X, et al (2009) Clara cell secretory protein is reduced in equine recurrent airway obstruction. Veterinary Pathology 46: 604–613. 19276063 10.1354/vp.08-VP-0255-B-FL

[25] Singh G, Singh J, Katyal SL, Brown WE, Kramps JA, et al (1988) Identification, cellular localization, isolation, and characterization of human Clara cell-specific 10 KD protein. Journal of Histochemistry and Cytochemistry 36: 73–80. 3275712 10.1177/36.1.3275712

[26] Montefort S, Baker J, Roche WR, Holgate ST (1993) The distribution of adhesive mechanisms in the normal bronchial epithelium. European Respiratory Journal 6: 1257–1263. 8287940

[27] Heijink IH, Brandenburg SM, Noordhoek JA, Postma DS, Slebos DJ, et al (2010) Characterisation of cell adhesion in airway epithelial cell types using electric cell-substrate impedance sensing. European Respiratory Journal 35: 894–903. 19741028 10.1183/09031936.00065809

[28] Devereux TR, Fouts JR (1980) Isolation and identification of Clara cells from rabbit lung. In Vitro 16: 958–968. 7005078 10.1007/BF02619334

[29] Massey TE, Geddes BA, Forkert PG (1987) Isolation of nonciliated bronchiolar epithelial (Clara) cells and alveolar type II cells from mouse lungs. Canadian Journal of Physiology and Pharmacology 65: 2368–2372. 3329569 10.1139/y87-375

[30] Oreffo VI, Morgan A, Richards RJ (1990) Isolation of Clara cells from the mouse lung. Environmental Health Perspectives 85: 51–64. 2200669 10.1289/ehp.85-1568317 PMC1568317

[31] Chichester CH, Philpot RM, Weir AJ, Buckpitt AR, Plopper CG (1991) Characterization of the cytochrome P-450 monooxygenase system in nonciliated bronchiolar epithelial (Clara) cells isolated from mouse lung. American Journal of Respiratory Cell and Molecular Biology 4: 179–186. 1991074 10.1165/ajrcmb/4.2.179

[32] Belinsky SA, Lechner JF, Johnson NF (1995) An improved method for the isolation of type II and Clara cells from mice. In Vitro Cellular and Developmental Biology Animal 31: 361–366. 7543342 10.1007/BF02634285

[33] Walker SR, Hale S, Malkinson AM, Mason RJ (1989) Properties of isolated nonciliated bronchiolar cells from mouse lung. Experimental Lung Research 15: 553–573. 2767004 10.3109/01902148909069618

[34] Martin J, Legg RF, Dinsdale D, White IN (1990) Isolation of Clara cells from rat lung using flow cytometry. Biochemical Society Transactions 18: 664. 2276503 10.1042/bst0180664

[35] Leung EL, Fiscus RR, Tung JW, Tin VP, Cheng LC, et al (2010) Non-small cell lung cancer cells expressing CD44 are enriched for stem cell-like properties. PLoS One 5: e14062. 21124918 10.1371/journal.pone.0014062 PMC2988826

[36] Germano D, Blyszczuk P, Valaperti A, Kania G, Dirnhofer S, et al (2009) Prominin-1/CD133+ lung epithelial progenitors protect from bleomycin-induced pulmonary fibrosis. American Journal of Respiratory and Critical Care Medicine 179: 939–949. 19234103 10.1164/rccm.200809-1390OC

[37] Bertolini G, Roz L, Perego P, Tortoreto M, Fontanella E, et al (2009) Highly tumorigenic lung cancer CD133+ cells display stem-like features and are spared by cisplatin treatment. Proceedings of the National Academy of Sciences of the United States of America 106: 16281–16286. 19805294 10.1073/pnas.0905653106 PMC2741477

[38] Hegab AE, Kubo H, Fujino N, Suzuki T, He M, et al (2010) Isolation and characterization of murine multipotent lung stem cells. Stem Cells Dev 19: 523–536. 19848595 10.1089/scd.2009.0287

[39] Tompkins DH, Besnard V, Lange AW, Wert SE, Keiser AR, et al (2009) Sox2 is required for maintenance and differentiation of bronchiolar Clara, ciliated, and goblet cells. PLoS One 4: e8248. 20011520 10.1371/journal.pone.0008248 PMC2788414

[40] Tompkins DH, Besnard V, Lange AW, Keiser AR, Wert SE, et al (2011) Sox2 activates cell proliferation and differentiation in the respiratory epithelium. American Journal of Respiratory Cell and Molecular Biology 45: 101–110. 20855650 10.1165/rcmb.2010-0149OC PMC3145063

[41] Volckaert T, Dill E, Campbell A, Tiozzo C, Majka S, et al (2011) Parabronchial smooth muscle constitutes an airway epithelial stem cell niche in the mouse lung after injury. Journal of Clinical Investigation 121: 4409–4419. 21985786 10.1172/JCI58097 PMC3204843

